# A Holistic Review on Euro-Asian Lactic Acid Bacteria Fermented Cereals and Vegetables

**DOI:** 10.3390/microorganisms8081176

**Published:** 2020-08-03

**Authors:** Tolulope Joshua Ashaolu, Anna Reale

**Affiliations:** 1 Smart Agriculture Research and Application Team, Ton Duc Thang University, Ho Chi Minh City 758307, Vietnam; tolulopeashaolu@tdtu.edu.vn; 2 Faculty of Applied Sciences, Ton Duc Thang University, Ho Chi Minh City 758307, Vietnam; 3 Institute of Food Science, National Research Council, ISA-CNR, 83100 Avellino, Italy

**Keywords:** lactic acid bacteria, fermented foods, cereals, vegetables, Europe, Asia, beverages, starter cultures, rice, wheat

## Abstract

Lactic acid fermentation is one of the oldest methods used worldwide to preserve cereals and vegetables. Europe and Asia have long and huge traditions in the manufacturing of lactic acid bacteria (LAB)-fermented foods. They have different cultures, religions and ethnicities with the available resources that strongly influence their food habits. Many differences and similarities exist with respect to raw substrates, products and microbes involved in the manufacture of fermented products. Many of them are produced on industrial scale with starter cultures, while others rely on spontaneous fermentation, produced homemade or in traditional events. In Europe, common LAB-fermented products made from cereals include traditional breads, leavened sweet doughs, and low and non-alcoholic cereal-based beverages, whereas among vegetable ones prevail sauerkraut, cucumber pickles and olives. In Asia, the prevailing LAB-fermented cereals include acid-leavened steamed breads or pancakes from rice and wheat, whereas LAB-fermented vegetables are more multifarious, such as kimchi, sinki, khalpi, dakguadong, jiang-gua, soidon and sauerkraut. Here, an overview of the main Euro-Asiatic LAB-fermented cereals and vegetables was proposed, underlining the relevance of fermentation as a tool for improving cereals and vegetables, and highlighting some differences and similarities among the Euro-Asiatic products. The study culminated in “omics”-based and future-oriented studies of the fermented products.

## 1. Introduction

Globally, the choice and consumption of healthier and safe food products has steadily increased in the past few decades, emphasizing the diet–human health connectivity. In this sense, functional products are excellent food options, as they aim to improve life quality by preventing nutrition-related diseases [1]. A huge percentage of these functional products are usually produced by lactic acid bacteria (LAB) via fermentation.

Fermentation is a cheap food biotechnological process used worldwide, a tool that is helpful in the production of several functional foods and beverages. The pyramidal base for the production of traditional fermented foods is in the household, in which an enormous amount of microbial and raw matter inoculants are used in a haphazard manner [2]. The low cost of fermentation and LAB cultures have made it easier to ferment numerous substrates from vegetables, cereals, dairy-milk, fruits and starchy root crops in order to improve their nutritional status, reduce toxic and anti-nutritional elements, and improve the shelf life and sensory properties of foods [3]. Whether in a solid state or submerged state, fermentation, through acidification, alcoholization, proteolysis and amino acid conversions, leads to the production of health-promoting food products with desirable qualities like shelf-life, texture, taste, mouthfeel, flavor and color [4].

Europe and Asia have long and huge traditions in the manufacturing of several LAB-fermented foods and, in the last years, thanks to scientific progress and “omic” studies, the knowledge, safety and regulation of many fermented products have increased.

Trade between European and Asian countries accounts for about half of the global trade, and though intra-regional trade is four times higher than cross-regional trade, the Euro-Asian regions trade more between them than between any other regions of the world [5]. As significant as the Euro-Asian economic ties are to the world, their foods are of equal global importance. The use of wheat and olive oil prevails in European foods with sweet or salt tastes, but Asian foods often include rice, sesame oil and soy sauce with spicy, bitter or sour tastes.

In general, natural fermentations are performed by lactic acid bacteria and yeasts. These microorganisms often act in synergy. Yeasts play an important role in the production of numerous fermented beverages and leavened breads thanks to their ability to produce high levels of ethanol, carbon dioxide and highly desirable aromatic compounds, while lactic acid bacteria are particularly important in the fermentation of cereals, vegetables, meat and dairy products for their acidifying and proteolytic activity, and for their ability to produce aromatic compounds.

In this review, our interest has focused only on the study of lactic acid fermentation, which, although having a secondary role for some fermented products, is however decisive in defining the final quality of the products.

This review considers cereals and vegetable products obtained via lactic acid bacteria fermentation in Europe and Asia. The review includes the relevance of fermentation as a tool for improving cereals and vegetables, the concept of lactic acid bacteria fermentation and the Euro-Asian LAB-fermented cereals and vegetables list, highlighting differences and similarities among products. It further describes an outlook on prospective studies and considerations.

## 2. Fermentation as a Tool for Improving Cereals and Vegetables

Cereals are the main consumed staple-food in most parts of the world. Their derived products can also be consumed alongside popular vegetables to make exotic delicacies. Being rich sources of carbohydrates and mineral nutrients, cereals and vegetables proffer numerous sensory, nutritional and physiological potentials when fermented. Natural or spontaneous fermentation was used by our ancestors for the preparation and preservation of foods like cereals and vegetables, without having any knowledge of microbes involved in the process [6].

In Europe, Asia or in other parts of world, people still practice the old traditional method of preparation of breads or loaves without using any commercial strains of baker’s yeast [7]. Furthermore, perishable and seasonal leafy vegetables, radish, onions, cucumbers and young edible bamboo tender shoots are traditionally fermented into traditional edible products such as khalpi, cucumbers, oiji, hom-dong, sauerkraut, olives, and jiang-gua [8]. Over time, pickling various types of vegetables by fermentation has become common in order to elicit some distinctive tastes from the products.

A general classification of food fermentation includes different categories: (a) fermentations, producing textured vegetable protein as meat substitutes in legume/cereal mixtures; (b) high salt/savory meat flavored/amino acid/peptide sauce and paste fermentations; (c) lactic acid, alcoholic, acetic acid/vinegar, alkaline fermentations; (d) leavened breads/sour dough breads; (e) flat unleavened breads [9].

LAB fermentation enables the preservation of numerous cereals and vegetables, as well as the transformation of the raw material into a new product with unique sensorial properties, new flavors, aromas, better textures and enhanced nutritional value. During fermentation, raw substrates become colonized by autochthonous microorganisms that use their enzymes to digest the food components into acceptable final products to the prospective consumers [10,11].

Cereals, for example, lack the essential amino acids and some other vital compounds for proper nutrition, thus fermentation helps in releasing these compounds by decreasing the levels of carbohydrates and non-digestible polysaccharides and oligosaccharides in cereals, and increasing the essential amino acids’ production and bioavailability [12,13]. Polyphenols, phytates and tannins are non-nutrients decreased in the process of LAB fermentation [14,15], while the binding capacity, digestibility, absorption and solubility of minerals such as iron increase [16].

The use of a desirable starting culture for the making of fermented foods may prevent contamination, and helps retain consistent food quality [6]. Therefore, important criteria to be considered when selecting fermentative microbial species and strains for the production of functional foods and beverages include the safety of microbial species/strains, nutritional quality, protectiveness, broad fermentability, bioactivity and sensory qualities [2,17].

## 3. Lactic Acid Bacteria (LAB) Fermentation

Fermentation is a way to exploit the activities of microorganisms so as to preserve and transform raw materials into high-quality products. Lactic acid fermentation is the oldest and most popular way to improve the functionality, nutritional value, taste, appearance, shelf-life and safety of cereal foods, and reduce the energy required for cooking [18].

Examples of lactic acid-fermented products, i.e., products primarily fermented by lactic acid bacteria, include yoghurt, cheese, sourdough, olives in the western world, sauerkraut (fermented cabbage from eastern and central Europe), thai pickled vegetables (fermented cabbage from Thailand), sausages, Chinese steamed bun, kimchi (fermented and spiced Napa cabbage from Korea), fermented soybean and rice [19].

Responsible for this kind of fermentation are the lactic acid bacteria, non-spore forming, acid-tolerant bacteria classified as anaerobic, facultative anaerobic bacteria, aerobic or microaerophilic, catalase-negative, and Gram-positive with coccoid or rod-shaped cells, which may form chains, pairs or tetrads [18].

The new bacterial taxonomy classifies the lactic acid bacteria in the phylum *Firmicutes*, class *Bacilli*, order *Lactobacillales*, and refers to five families: *Lactobacillaceae* (which now includes the family *Leuconostocaceae*), *Streptococcaceae, Enterococcaceae, Carnobacteriaceae* and *Aerococcaceae* [20].

Lactic acid bacteria are among the most familiar bacterial groups to humans, since they are widespread in nature, occurring in numerous nutrient-rich habitats such as plant, soil, animals and humans. They can as well be found fermenting already consumed food products in the gut [21]. The isolation and identification of lactic acid bacteria from raw matrices (grains, crops) is naturally fermented foods (dairy products, cereals, fruits and vegetables) is well documented [8].

Many genera/species of microorganisms have been reported in relation to various fermented foods and beverages across the world.

The lactic acid bacteria associated with foods include species of the genera *Lactobacillus* (old classification), *Leuconostoc, Pediococcus, Lactococcus*, and *Streptococcus*, as well as the more peripheral *Aerococcus, Carnobacterium, Enterococcus, Oenococcus, Sporolactobacillus, Tetragenococcus, Vagococcus*, and *Weissella*.

Actually, scientists have recently reclassified the genus *Lactobacillus* into 25 genera, including the emended genus *Lactobacillus*, which includes host-adapted organisms that have been referred to as the *L. delbrueckii* group, *Paralactobacillus* and 23 novel genera: *Acetilactobacillus*, *Agrilactobacillus, Amylolactobacillus, Apilactobacillus, Bombilactobacillus, Companilactobacillus, Dellaglioa, Fructilactobacillus, Furfurilactobacillus, Holzapfelia, Lacticaseibacillus, Lactiplantibacillus, Lapidilactobacillus, Latilactobacillus, Lentilactobacillus, Levilactobacillus, Ligilactobacillus, Limosilactobacillus, Liquorilactobacillus, Loigolactobacilus, Paucilactobacillus, Schleiferilactobacillus* and *Secundilactobacillus* [20]. (In this review, we keep the names of the old classification of the genus *Lactobacillus* to avoid any confusion in the reader. However, the links for the appropriate name conversions are as follows: http://lactobacillus.ualberta.ca; http://lactobacillus.uantwerpen.be; http://lactotax.embl.de/wuyts/lactotax/).

LAB are heterotrophic microorganisms, and have high nutritional requirements for amino acids and vitamins, because they have lost much of their biosynthetic potential in the process of evolution. For this reason, they only develop in nutrient-rich environments.

They ferment carbohydrates to almost entirely lactic acid (homofermentation), or to a mixture of lactic acid, carbon dioxide and acetic acid and/or ethanol (heterofermentation). Other compounds, such as diacetyl, acetaldehyde and hydrogen peroxide, are also produced. These compounds contribute to the flavor and texture of fermented foods, and may also contribute to the inhibition of undesirable microbes [22]. In addition to organic acids, LAB synthesize a wide range of beneficial metabolites, such as antifungal and anti-mycotoxigenic agents, bacteriocins and nutraceutical products.

The richness and diversity of microbial species, with their relative abundances and interactions, determine the establishment of distinctive and unique characteristics of each fermented product.

Despite common features, however, many differences exist with respect to substrates and products, and the types of microbes involved in the manufacture of fermented foods and beverages produced globally.

## 4. European LAB-Fermented Cereals and Vegetables

### 4.1. Fermented Cereals

In European countries, the fermentation of cereals is used mainly for the obtainment of bakery products (typical breads and sweet traditional leavened cakes) and for different beverages, including the ales and beers of northern Europe, the lagers of central and eastern Europe and numerous low or non-alcoholic beverages that contain a maximum of 0.5% volume of ethyl alcohol [23].

The most common and uncommon European products made from grains and obtained mainly by lactic acid fermentation are observed in Table 1. Regarding bakery products, most cereals like wheat, rye, barley, spelt and maize are fermented by natural fermentation (sourdough) or by adding commercial baker’s yeast to leaven the doughs [24].

In Europe, countless different types of “Breads”, including traditional typical breads [25,26,27,28,29,30,31,32], “Pizza” [33] and artisan sweet cakes as Italian “Panettone”, “Pandoro” and “Colomba”, are made by use of sourdough from mainly wheat and rye. In Portugal, maize flour is used in combination with rye flour to produce *broa*, a home-baked sourdough bread, still following ancient protocols [34,35,36]. In northern Europe, sourdough is employed especially in the baking process of rye flour, for example in Germany, the Baltic states and Russia [37,38,39]. Minor breads are produced by minor cereals such as spelt, maize or buckwheat [40,41,42]. Teff and teff sourdoughs are promising ingredients for bread production [43].

Sourdough is obtained by the fermentation of autochthonous microorganisms (bacteria and yeast) that are naturally present in the environment, or in raw materials or ingredients, conferring each sourdough a unique characteristic. Sourdoughs are characterized by more than 50 different species of lactic acid bacteria, but it is very likely that a non-identifiable and perhaps new sourdough LAB species exists [44].

In Europe, among fermented cereal products other than bakery products, different low and non-alcoholic cereal-based beverages have been developed. The development of cereal-based fermented beverages is in response to the growing prevalence in consumer vegetarianism, lactose intolerance, cholesterol content and economic reasons that are associated with dairy products. In fact, cereal-based beverages fermented with LAB could be a perfect alternative for people who are allergic to milk proteins [45]. Such products include “kvass” and “boza”, which are cereal-based beverages traditionally made from fermented barley and rye malt, rye flour and millet flour.

“Kvass” is a cereal-based beverage traditionally made from fermented barley and rye malt, rye flour and stale rye bread that is mainly produced in eastern countries such as Russia and Poland, and Baltic countries, especially Lithjuania and Latvia [57]. It is a non-alcoholic beverage that contains 1% or less alcohol. The microflora of kvass fermentation is normally composed of yeasts (*Saccharomyces cerevisiae*) and LAB such as *Lactobacillus casei* and *Leuconostoc mesenteroides* [58,59]. As it is a product of lactic and alcohol fermentation, it does not require pasteurization and, therefore, contains LAB, which are useful microflora for human health. Therefore, kvass can be considered as a probiotic beverage. Different authors showed that Kvass eliminates flatulence, hyperacidity and other digestive disorders and, at the same time, has a positive effect on metabolism, increases the bioavailability of calcium and other minerals, and possesses antioxidant properties [45].

Another traditional ancient fermented cereal, produced in different East European, countries is “Boza”, a cereal beverage that is obtained from the fermentation of whole grain or flour of different cereals (usually millet, barley, oats, maize, rye, wheat or rice), but boza of the best quality and taste is made of millet flour. It is very popular in Turkey, Albania, Bulgaria, Macedonia, Montenegro, Bosnia and Herzegovina, and parts of Romania and Serbia, as well as in South Russia. It is a viscous low-alcoholic drink with a pleasant sweet taste and slightly acid flavor, varying in color from creamy-white and beige to light brownish [48].

Due to boza’s pleasant flavor and taste and high nutritional value, it has become a very popular drink consumed daily by people of all ages. The compositional variations of boza samples result from the utilization of different types and amounts of cereals as raw materials, and uncontrolled fermentation conditions [49].

The microbiota responsible for the fermentation of Boza seem to be particularly heterogeneous, including both homo- or hetero-fermentative LAB (*L. plantarum, L. brevis, L. fermentum, Leuc. mesenteroides, L. casei, L. acidophilus,* etc), which provide the acid character of boza, and yeasts responsible for alcohol fermentation [49,50,51,52]. It is a highly nourishing drink, which is easily digestible, gratifying in taste and fragrance, and exerts beneficial effects on human health due to its probiotic microbial content.

“Trahanas” is one of the most popular fermented milk-cereal products from Greece and Cyprus, and it is named ‘Tarhonya’ or ‘Talkuna’ in Hungary and Finland, respectively. It is produced during summer, mainly from whole fresh ewes’ and goats’ milk or a mixture of them. Sometimes, instead of milk, a pulp of vegetables is used. For the production of trahanas, fresh milk is allowed to be acidified for some days either spontaneously or by adding a culture of yoghurt [46]. Fermentation of trahanas is important for the development of the product’s flavor and aroma, which are mainly produced by *Streptococcus lactis, Str. diacetylactis, Leuc. cremoris, L. lactis, L. casei, L. bulgaricus* and *L. acidophilus* [47]. Trahanas is stirred every day until it reaches the desired acidity. It is then heated, followed by the gradual addition of ground wheat and salt. Sometimes eggs are also added and the final product is called “sour trahanas with eggs”. Methods for the preparation of such products vary from place to place, but cereals and fermented milk are always the two major components [46]. Trahanas is a product with high dietary fiber content mainly due to the wheat used for its production. Greek ‘Trahanas’ is a very nutritive food as it is a good source of proteins, minerals and other nutrients, and is used largely for feeding people.

Another wheat bran-based fermented beverage is “Borş”, traditionally prepared in Romania. It is used to give a sour taste to traditional soups or consumed as a refreshing drink. It has many health benefits, especially due to the high vitamin and mineral contents. Information about Romanian borş is scarce, mainly restricted to travel guides and cooking books, as this fermented beverage did not receive any scientific attention before this decade.

Romanian borş was investigated for the first time regarding its microbiological and physicochemical characteristics by Grosu-tudor [56], who showed that Romanian borş fermentation was mainly carried out by lactobacilli, among which *L. amylolyticus* was the most frequently found LAB species. Other lactobacilli frequently found were *L. fermentum, L. plantarum, L. casei* and *L. buchneri*. The fermentation of wheat bran during the production of borş leads to increased concentrations of available amino acids and phenolic compounds. Borş is a good revitalizing product, which was always used for its perceived beneficial effects on respiratory diseases, digestive problems (indigestion, vomiting), liver and bile diseases, and even cancer treatment.

There are many other cereal-fermented products typical of the traditions of the East European countries, still little studied. Historically, several low-alcoholic drinks were made in Estonia such as “Taar” and “Kali”. “Taar” is made of rye and barley, rarely also oats. Grains are milled into flour and mixed with boiled water. The beverage has to turn sour for 2–3 days, and the resulting drink is “taar”. To prolong the preservation of taar and to add specific taste, aromatic species (like *Origanum vulgare* and *Ledum palustre*) are added during the fermentation process [54].

“Kali” is another alcohol-free drink from Estonia. Its technology is similar to that used to make taar, but it is made of malted cereals and produced after the beer was already made. It’s like a mix between a beer and a cola, with a sour-sweet taste, a natural fizz, and very low alcohol content [23].

Furthermore, there are other uncommon (and endangered) preparations from fermented cereals. Different fermented dishes and drinks made of oat were widely consumed as regular meals in Estonia until the end of 19th century, and sporadically until the middle of 20th century, but nowadays their popular use is limited to special groups trying to restore national food heritage. For example, oat seeds were macerated in warm water, left to ferment for a few days, and then the milky water was squeezed out and the resulting sour liquor, called “kaera kiesa” or “kaerapiim”, was drunk on the side of the meal [54].

A bit of a different beverage, called “kile”, was made of oat flour mixed with water; it was let to stay in warmth for a night. This filtered sour beverage was consumed instead of sour milk on the side of the meal. If the filtrate was boiled, it became a kind of gruel, which was also called kile, but also “kiisel” or “kisla”, and eaten hot with butter or fat, or later as a cold jelly. Similar gruels (also similarly named) were prepared from rye or from rye and potatoes [55].

### 4.2. Fermented Vegetables

In Europe, a total of 21 different vegetables are fermented, in addition to an unspecified number of variably composed vegetable blends and fermented vegetable juices [87]. Most vegetables or their juices will undergo a spontaneous lactic acid fermentation when conditions are anaerobic and moisture levels, salt concentrations and temperature are appropriately adjusted so that the lactic acid bacteria have a competitive advantage [88].

Most fermented vegetables typical of European countries such as pickles, capers, cucumber, sea fennel, turnip shreds and hardaliye are still fermented on a small scale, either in the home as side dishes or as ingredients. Instead, “table olives” and “sauerkrauts” are the major fermented vegetables produced in western countries, are of significant commercial importance and will be treated in depth in this paragraph (Table.1).

“Table olives” are a traditional food of the Mediterranean countries (Spain, Greece and Italy, mainly in Sicily), with many centuries of history. They are prepared with fruits obtained from cultivated *Olea europaea* subsp. *europaea* var. europaea trees, and their production has expanded to many countries around the world [60,61,89].

After harvest, olives are inedible due to bitterness, and have to be processed to remove the fruit bitterness by hydrolysis of the phenolic compounds, especially oleuropein.

Globally, there are three main methods of obtaining table olives: Spanish- or Sevillian-style green olives in brine; Californian-style black ripe olives and Greek-style natural black olives in brine. However, there are many other traditional table olive elaboration recipes, which are less known in the international market [90] and constitute niche products, such as table olives produced in Sicily, that do not undergo preliminary treatment in sodium hydroxide (lye) before fermentation [91].

All methods rely on the autochthonous or indigenous microbiota for fermentation, with LAB and yeasts as the primary organisms. Lactic acid bacteria are involved in the fermentation of Spanish-style treated olives, while yeasts are responsible for the fermentation of Greek and Californian processed black olives, with LAB representing a small proportion of the total microflora [92].

Lactic acid bacteria, which convert fermentable sugars to lactic acid and other organic acids depending on their metabolic pathways, are the most important group of bacteria in olives [62]. LAB fermentation of olives reduces their pH, provides microbiological stability and extends their shelf life.

Together with LAB, yeasts play a substantial role in fermented olive production, contributing to the determination of their aroma and taste due to the production of desirable metabolites and volatile compounds, while at the same time they enhance the growth of LAB and degrade phenolic compounds [93,94].

Different studies highlighted that the use of selected starter cultures, and more particularly the use of autochthonous LAB strains isolated from a spontaneous fermentation of the respective olive cultivar, could lead to a more controllable fermentation resulting in a product of high and constant quality, which is safer for the consumers and has a longer lifetime, and this process also influences the volatile organic compounds (VOC) profiles of olives [95,96].

Like table olives, “Sauerkraut”, primarily from cabbage, is one of the most well-known varieties of fermented vegetables associated with central and eastern European cultures, though it can be found in western European cuisine as well. For this product, the crucial importance of LAB fermentation has also been confirmed.

In fact, though the microbial composition of sauerkraut can vary during the initial stages of fermentation, appropriate fermentation conditions, such as temperature and relative ingredient concentration, ensure that LAB are the dominant microorganisms in the final fermented product. Zabat et al. [67] highlighted that although LAB exhibit low relative abundance in the starting ingredients, and in the production facility environment, they are able to dominate the fermented sauerkraut, confirming the high capability of LAB to cope with the strong selective pressures of high salinity and acidity during fermentation with respect to other microorganisms.

LAB guarantee the success of cabbage fermentation by the production of organic acids, bacteriocins, vitamins and flavor compounds responsible for many of the characteristic sensory qualities of the final fermented products, including extended shelf life. Moreover, numerous LAB isolated from sauerkraut were found to be probiotic or putative probiotic cultures [68,97].

Several other varieties of vegetables (such as carrots, French beans, marrows, artichokes, capers and eggplants), which are mainly cultivated in southern Italy or, more generally, in the Mediterranean area, may increase their safety, nutritional, sensory and shelf-life properties through lactic acid fermentation under standardized industrial conditions [98].

Among these minor fermented products occurring in the European countries are “caper”, ”sea fennel”, ”turnip shreds” and ”cucumber”, regarding which few scientific studies have yet been carried out, focusing on their microbial communities and population dynamics during lactic acid fermentation.

For example, “Caper” berries are the fruits of *Capparis species* (mainly *Capparis spinosa* L.), a Mediterranean shrub cultivated for its buds and fruits.

The main producers of fermented caper berries are Mediterranean countries, especially Greece, Italy, Turkey, Morocco and Spain, and the final products are exported mainly to central European countries, the United States and the United Kingdom as a delicatessen product [99]. The fermentation of caper fruits is often done by traditional artisanal ways. Fruits are harvested during June or July, immersed in tap water, and subjected to spontaneous lactic acid fermentation for ca. 5–7 days at ambient temperature, which may markedly vary from 23 °C to 43 °C. Subsequently, fermented capers are placed into brine and distributed for consumption. *L. plantarum* is the main species, which is isolated from the brine of capers [100].

“Sea fennel”, known as maritime rock (*Crithmum maritimum* L.), is a salt-tolerant plant, widely grown in maritime rocks and more rarely sandy beaches in Mediterranean countries and Atlantic coasts. This plant has several usage areas such as culinary, medicine and cosmetics because of the content of the nutrients and phytochemicals. In the Mediterranean region, their leaves are consumed fresh or fermented as salad or with yoghurt [65].

Fermented “turnips” are a traditional food in both Asia and Europe. In Korea, turnips are used in a form of kimchi whereas, in Europe this is a traditional fermented vegetable produced mainly in Germany (known as sauerruben) and in the northeast Italian region (known as Brovada).

Brovada is obtained by natural fermentation of turnips (*Brassica rapa*). It is similar to sauerkraut, but in some aspects the technology of production is different. Turnips are cleaned and put in vats, alternating them with layers of grape skins. Before covering the vat, a mix of water and salt or water only is added. The fermentation is a spontaneous process caused by the microorganisms present on the various components (turnip and grape skins) and goes on for about 30 days. After that, the turnips are extracted from the vats, peeled, cut into pieces and wrapped up in plastic bags ready for selling. The product is generally eaten after cooking, but sometimes even raw [66].

Another fermented vegetable, produced both in Asia and Europe, is the “Cucumber”. Considering that fresh cucumbers expire relatively fast, they are most frequently eaten as fermented pickles. Fermented pickles are products stabilized with salt and lactic acid, which are produced by lactic acid bacteria fermentation. They can be produced in different ways. In the northern parts of Europe, especially in Poland, cucumbers are fermented in the presence of dill weed, garlic, blackcurrant and oak leaves and horseradish at a final brine concentration of 3–4%. Spices give the products a desirable flavor, and antimicrobial substances present in them prevent mold and yeasts growth. The cucumbers are not desalted and they have to be stored at 0–5 °C [101]. *Lactobacillus* spp., *Leuconostoc* spp. and *Pediococcus* spp. are the main LAB naturally present on the cucumber surface and responsible for fermentation [69].

Another non-alcoholic fermented beverage produced in a traditional way in Thrace, the European part of Turkey, is “Hardaliye”. Hardaliye is a lactic acid-fermented traditional beverage produced from grape juice and crushed grapes, with the addition of different concentrations of whole/ground or heat-treated mustard seeds and sour cherry leaves [63]. The predominant LAB species found in hardalyie are *L. paracasei* subsp. *paracasei* and *L. casei* subsp. *pseudoplantarum*, followed by *L. pontis*, *L. brevis, L. acetotolerans, L. sanfranciscensis* (formerly known as *L. sanfrancisco*) and *L. vaccinostercus* [64].

## 5. LAB-Fermented Cereals and Vegetables of Asian Origin

### 5.1. Fermented Cereals

In Asia, especially in China, Japan, Korea and other Far East regions, where pastoral agricultural practices and animal husbandry are more limited, fermented foods that evolved were based more often on rice and grains, soybeans, vegetables and fish as the primary substrates [102].

Lactic acid fermentation of cereals is a long-established processing method and is used in Asia for the production of foods in various forms, such as (semi)-solid cooked dough, gruels, porridge and liquid beverages.

Although differences exist between countries, processing the cereals may involve soaking, heating, mashing, filtering and backslopping. In general, cereals are soaked in water for a couple of days to soften the grains for easier crushing and wet-milling into slurry, from which hulls, bran particles and germs can be removed by sieving procedures. During the slurring or doughing stage, which lasts for 1 to 3 days, mixed fermentations including lactic acid fermentation take place [103].

In Asia, the main fermented cereals are millet, maize, barley, oats, rye, wheat, rice and sorghum, easily fermented by naturally occurring microorganisms. In particular, rice and soybean are the commonest foods in Asia, and are popularly fermented. In Table 1 are the main popular LAB- fermented cereals of Asian origin.

Rice noodles, called “Nombonchock” in Cambodia or “Khanom jeen” in Thailand, are made by lactic acid bacteria fermentation, which gives them a low fermented odor, flexibility and smoothness [71,72,73,104,105].

For these rice noodles, broken rice grains, usually stored/fermented over a six-month period, are naturally fermented for a few days, wet milled, and then left to precipitate as rice flour, which is then placed in a cotton bag under an applied weight for excess water removal. The fermented rice flour is then pre-cooked, kneaded and extruded into noodles in boiling water, and is immediately cooled and dried [106].

Fermentation in nombonchock/khanom jeen is achieved by lactic acid bacteria acting on the lipid, protein and ash contents to enhance the stringy mouth feel of the noodles, and a number of volatile compounds such as 3-methylbutanoic acid and diacetyl, 2-methylpropanoic acid, are synthesized by the metabolic activities of these microorganisms, which impart a characteristic flavor to nombonchock/khanom jeen [107].

“Amazake” is a Japanese fermented sweet food beverage prepared from rice.

There are two types of amazake depending on the preparation method: a low-alcoholic drink made with lees left from sake (sakekasu amazake) and a non-alcoholic drink made with koji rice that can be enjoyed by everyone including small children (koji amazake, in Japanese).

Koji rice is cooked rice that has been inoculated with *Aspergillus oryzae*, a mold that is widespread in Japan. It is at the heart of many different Japanese ferments. It is used to make miso, sake, amazake, rice vinegar, soy sauce and mirin [108]. In either of the preparation methods, the mixture is allowed to ferment for 6–8 h, thereby leading to saccharification and conversion of the rice starches into glucose and oligosaccharides. The rice’s vitamins and amino acids also become converted into an easily digestible form. There is no dairy or gluten in amazake, and it may serve as a natural sweetener in the production of other traditional drinks.

Amazake is a sake precursor and known for its health benefits, including weight loss and stress relief, positive skin effects, innate immunestimulating activity, antihypertensive effects, cholesterol lowering effects, and tyrosinase-inhibiting activity [108,109,110,111]. Amazake is rich in functional ingredients including vitamin B, glucoses and amino acids, as well as fermentation products of rice malt fungi [112]. The non-alcoholic amazake is used as a baby weaning formula due to its ease of digestion. Recently, *L. sakei* strains (UONUMA 1, 2 and 3) were reported as crucial for the promotion of amazake’s nutritional, sensorial and functional properties [113].

Further, “Com me” is a sour rice fermented paste, a Vietnamese traditionally fermented food. It is a sour fermented paste made from overcooked wet rice fermented in clean and tightly closed containers for 7–10 days. Sometimes, the fermented product from a previous batch is added to the present batch to expedite the souring process (backslopping). During fermentation, rice changes from the whole grain state to a macerated product, and finally to a milky cream substance with a light sour aroma. After fermentation, the product is crushed and sieved. Com me is used as a souring agent during cooking and gives a meal a specific flavor and sour taste [76]. The presence of *L. plantarum, L. paracasei, L. casei* and *L. acidophilus* species was found in “Com me” samples from provinces along the Mekong river delta (South Vietnam).

Another traditional Asiatic cereal-fermented food is “Tarhana”. It is a traditional Turkish food prepared by mixing wheat flour, yoghurt and some vegetables and spices, followed by fermentation, drying and grinding [114].

It is a good source of nutrition for children and the elderly, since it provides increased digestibility and nutritional value due to its high bioactive proteins and prolonged fermentation process [16,115,116]. Generally, tarhana mix is fermented by the natural microbial flora of the flour and other ingredients included. The dough of Tarhana is prepared after 1–7 days’ lactic and alcoholic fermentation. Thereafter, the dough is sun-dried and ground. Traditionally, the grounded dough of Tarhana is used to make soup. The characteristic taste and flavor of Tarhana are derived from lactic acids, ethanol, carbon dioxide, and some organic compounds produced by LAB and yeast. Kivanc et al. [78] found various species of yeast and lactic acid bacteria occurring in Tarhana during natural fermentation. They observed that *Lactococcus lactis* spp. *lactis*, *Leuc. mesenteroides*, *L acidophilus, Enterococcus durans, Pediococcus* spp., *L. delbrueckii* ssp. *lactis* and *L. paracasei* bacteria played important roles in the fermentation of Tarhana dough. Furthermore, *Kluyveromyces marxianus*, *Yarrowia lipolytica*, *Pichia membranaefaciens*, *Pichia mexicana, Pichia angusta, Debaryomyces hansenii, Candida sorboxylosa, Candida fluviatilis* and *Saccharomyces cerevisiae* were identified during Tarhana fermentation.

There are several products similar to Tarhana in European countries. They are known as Thanu in Hungary, Trahanas in Greece and Talkuna in Finland.

Further, “Boza” is a traditional Turkish beverage made by the fermentation of millet, cooked maize, wheat or rice semolina/flour. Two different types of fermentation occur simultaneously during boza fermentation. In one, alcohol fermentation produces carbon dioxide bubbles and increases the volume. The second is lactic acid fermentation, which produces lactic acid and gives the acidic character to boza. In either of the two fermentative processes, the cereals are boiled for 1–2 h, cooled and strained. Sugar is then added before fermentation for up to 24 h. Apart from the yeasts present, *Lactobacillus, Micrococcus, Leuconostoc* and *Streptococcus* genera are the commonest LAB used [117].

Boza is a thick liquid, pale yellow in color with a characteristic acidic–alcoholic odor, and it is a favorite drink, particularly on cold winter nights. It is a healthy and nutritious beverage, and its lactic acid content has positive effects on digestion and intestinal flora [21,118]. A previous report on an improved sensory version of boza was associated with *Leuc. mesenteroides* and *L. confusus* as the main fermentative LAB species [53].

There are also similar beverages to boza that are produced in East European countries, known as braga or brascha, as well as in the Balkan region, also known as boza.

Tape ketan (fermented glutinous rice) and tape singkong (fermented cassava) are traditional Indonesian foods produced by fermenting carbohydrate sources using ragi as the starter culture [79]. Mold, yeast and bacteria have their own roles in developing the characteristics and flavor of tape. The LAB found in tape are *Weissella* spp, *Pediococcus pentosaceus* and *Enterococcus* spp [119].

Tape ketan is a sweet alcoholic drink similar to the sweet Japanese sake. The alcoholic content could be as high as 10% and largely depend on fermentation time. Its production involves boiling glutinous rice, cooling and inoculation with crushed ragi yeast cake or other desired LAB, and then fermented for 1–3 days.

Selroti is a Nepali sweet rice bread, mostly made and eaten during the Hindu festivals of Dashain and Tihar that take place in Nepal and some parts of India. The preparation involves the addition of milk, water, cooking oil, sugar, ghee, butter, cardamom, cloves, bananas and other flavors to a semi-liquid rice flour dough, which are thoroughly mixed, allowed to ferment for between 4 and 24 h, and fried.

Yonzan and Tamang [80] reported *Leuc. mesenteroides, E. faecium, Pc. pentosaceus* and *L. curvatus* as the predominant LAB found in selroti, alongside other yeasts. They also reported that *Leuc. mesenteroides* is the most prevalent LAB in selroti batters.

Moreover, in different Asiatic countries, wheat is widely used to produce sourdough-based products. In fact, dough for the various flat breads (e.g., naan, pita and kirsa) made in parts of Asia, as well as dough for the production of steamed bread in East Asia, is fermented in a process resembling that used for sourdough bread in Europe [120]. Furthermore, einkorn sourdough is commonly used to produce tasty breads [77].

The Chinese steamed bun also called baozi is a traditionally fermented wheat product originating from China, but it is consumed in many other countries in Asia. The bun has been a staple food for more than 2000 years [121], and is broadly classified into two types. The first needs starter dough for fermentation, just like sourdough, while the second type requires baker’s yeast. The production of Chinese steamed buns from starter doughs requires the addition of wheat flour and water to the starter dough, and fermentation for 16 to 20 h. The fermented dough is mixed with other ingredients (flour, water, sugar and leavening agents), shaped as a bun, stuffed and then steamed [75]. The steaming process makes it differ from oven-baked sourdough or other bread products in Europe, which gives it a thin smooth white skin, rather than the thicker brown crust of western oven-baked breads. More than thirty LAB were reportedly isolated from this fermented cereal product, which alter its taste, texture, and nutritional and functional qualities [75].

### 5.2. Fermented Vegetables

Asians generally are lovers of fermented food products, particularly Southeast Asians.

Fermented vegetables such as Inziangsang, Kimchi, Pak-Gard-Dong, Khalpi, Dakguadong, Jiang-gua, Burong mustala, Gundruk, Ca muoi, Soidon, Suan-tsai, Sayur asin, Paocai, Sauerkraut, Nozawana-Zuke, Yan-jiang and Yan-tsai-shin are habitually consumed by Thai, Indian, Chinese, Vietnamese, Japanese, Korean and Taiwanese people [6,82,122,123,124,125,126,127,128,129,130].

Most traditional preparatory and fermentation processes are kept secret, and are passed on from generation to generation in tribes and castes in different provinces, while many of them are made at the home scale using backslopping [131]. Different kinds of vegetables are also fermented, mainly by mixing with salt for a few hours and then adding rice-wash water (to accelerate fermentation time and to get an attractive color), to be eaten the following day [105].

Most of the vegetable fermentations occur spontaneously and provide conditions that favor the growth of LAB and inhibit undesirable microorganisms [105]. Lactic acid bacteria fermentation disrupts the mechanism of spoilage microorganisms via the oxidation of carbohydrates to carbon dioxide, alcohol and organic acids [132]. This in turn helps in the prevention of some diseases like diarrhoea and cirrhosis, while the antioxidants in fermented vegetables can aid in eradicating harmful free radicals that act in the developmental process of degenerative diseases [16,130].

The fermented vegetables in Asia are numerous. In this review, just the most widely consumed will be described in depth (Table.1). In Korea, a popular lactic acid bacteria fermented vegetable called “Kimchi” is widely consumed. It is traditionally prepared by immersing cabbage in a pool of vast ingredients including fish sauce, spices and radishes. This is followed by fermentation with various strains of LAB and other microorganisms, and it is consumed raw globally [16,133]. Kimchi is reported to reduce hte risks associated with carcinogenesis, atherogenesis, oxidation, tumors, bacterial infections, obesity, inflammation, mutagenesis and cancer, in addition to slowing aging, lowering cholesterol, stimulating the immune system, and containing probiotics [16,134].

“Sinki”, a non-salted fermented radish taproot product, is traditionally consumed as a base for soup and as a pickle in some north-eastern states of India, in Nepal and a few places in Bhutan [85]. Sinki is a preserved Nepali fermented vegetable prepared from radish tap roots. It usually takes about a month of bacterial curing, followed by drying in the sun and storage. Production involves wilting the radish tap roots, cutting off the leafy tops and shredding the radish tap-root sections. If it is for pickling purpose, the radish is just cut up, and left to ferment in a 2–3 foot hole above a small fire. The fire is later quenched after enough warmth is attained, replacing the bottom with bamboo, straw and further fortifications such as more vegetation, boards, rocks and mud to create a mostly air-tight barrier. Fermentation takes 20–30 days, followed by sun-drying, before it can be eaten.

If it is consumed as a pickle, drying is not required; rather, sinki is mixed with spices and bottled. Tamang and Sarkar [85] reportedly found numerous strains of *L. plantarum, L. brevis* and *L. fermentum* to be the predominant LAB in the fermentation of sinki.

Fermented cucumbers are produced both in Europe and Asia. Fermented cucumbers are turned into a product called jioang-gua in Taiwan, khalpi in Nepal and India, paocai in China, or oiji in Korea, and are referred to as pickles in many parts of the United States, Europe (Russia, Ukraine) and Canada [86]. Most cucumbers are fermented in a salt solution. Cucumber fermentation relies on the presence of naturally-occurring LAB or, in some cases, inoculum from a pure starter culture.

Thai pickled vegetables (Phak-dong) fermented with *B. siamensis* B44v are also consumed in Thailand [135]. These traditional fermented foods are obtained by the fermentation of naturally occurring LAB, such as *L. casei, L. plantarum, L. brevis, L. pentosus, L. fermentum, L. paracasei, Weissella koreenis, W. confusa, W. cibaria, Leuc. kimchi*, *Leuc. mesenteroides, L. fallax, Enterococcus* and *Pediococcus* species [6]. The lactic acid bacteria-mediated fermentation of vegetables is a standard method for the preservation and advancement of their dietary and sensory characteristics [70].

“Tsukemono” refers to Japanese pickled vegetables that are traditionally fermented with salt and seasonings for the purpose of shelf-life-extension, as well as taste and flavor enhancements. Preparation involves washing and direct pickling in nukadoko (a mix of rice bran, salt, seasonings and water), with proper mixing to effect anaerobic fermentation balance. Microbial growth activation takes more than 7 days, and the microbes that are involved in the fermented vegetables are mainly lactic acid bacteria, including *L. brevis, L. namurensis, L. acetotolerans, L. sakei, L. curvatus, L. collinoides, L. fructivorans, L. casei, L. casei subsp. pseudoplantarum, L. delbrueckii, L. fermentum, L. plantarum, L. citreum, Pc. pentosaceus* and *Pc. damnosus* [84].

In addition, “Miang” is a fermented Thai vegetable. The production involves steaming Thai tea leaves, which are then wrapped tightly and packed in a container. The leaves are pressed tightly under a heavy weight covered with banana leaves and fermented for 4–7 days, or even up to a year depending on the maturity of the tea leaves [81].

The fermented product is similar to Myanmar’s lak hpak, and is famous in northern Thailand. It is usually consumed together with peanuts, ginger, garlic or roasted coconut sliced as a side dish [81]. The main microorganisms involved in the fermented products include the genera *Lactobacillus, Streptococcus* and *Leuconostoc*, as well as several other members of the LAB group.

## 6. Prospective Studies and Considerations

Generally safe, high-quality and acceptable ethnic and traditional fermented cereals and vegetables are important because they have eminent roles in the traditions, histories, and social and economic activities of Euro-Asian cultures and peoples. The fermented food products may be significantly improved using commercial starter cultures such as LAB, except that current molecular and technological developments provide much better and faster prospects, with increased accuracy and sensitivity. The culture-independent methods are critical for investigating the metagenomic compositions of diverse and complex microbial communities within fermented food products. Available databases serve as match references to microbial DNA sequences, ascertaining individual microbes’ identity and potential [2]. A combination of DNA sequencing with bioinformatics provides useful identity characterization of an organism in a more rapid and accurate way.

Metagenomics and metatranscriptomics, although still underexploited, promise to uncover the functionality of complex microbial consortia directly in the food matrix, and understand microbial behavior in response to different process conditions [136]. In metagenomics or metatranscriptomics studies, no PCR is performed, and total DNA or cDNA is sequenced. Metagenomics analysis allows the obtaining of the taxonomical composition of the community, and the abundance of all microbial genes. The potential activities of the microbial communities are highlighted, because a specific gene may not be really expressed in that condition, or because DNA may arise from dead or metabolically inactive cells. In order to identify the genes actually expressed in a food sample, RNA sequencing (RNA-seq) is the most appropriate path to take, which is what happens in metatranscriptomics [136].

Metatranscriptomics is currently being considered as an option for doing an in-depth investigation into gene expressions of fermented foods’ microbial communities. The results may be coupled with other ‘omics’-based approaches, including proteomics and metabolomics, for a much deeper insight into the interactions of the microorganisms in association with the organoleptic and physicochemical properties of fermented foods [2].

Using a combination of 16S rRNA sequencing with tricine-sodium dodecyl sulfate-polyacrylamide gel electrophoresis (tricine-SDS PAGE), certain fermented foods from Cambodia were determined to contain bacteriocin-producing LAB [74], and the bacterial diversity of kefir drinks from Bosnia and Herzegovina was obtained through rRNA gene amplicon sequencing by Garofalo et al. [137]. Denaturing gradient gel electrophoresis (DGGE) methods are now used to screen and identify fermented foods’ microbiota, such as the LAB in Italian sourdoughs [25] and boza [48]. Pla-som and nem chua, Thai sour fermented fish and Vietnamese pork sausage, respectively, were investigated with these techniques [138,139].

Ribosomal DNA sequencing, and molecular typing by random amplified polymorphic DNA (RAPD) techniques, are other methods used in the determination of the LAB and other bacterial strains in fermented foods such as nham [105] or boza [48].

Similarly, restriction fragment length polymorphism (RFLP) analysis of 16S ribosomal DNA (rDNA) is a potential tool for the overall screening of the LAB-fermented cereals and vegetables in Euro-Asia and globally, as it was successfully employed in the identification of LAB used as starter cultures for sensorial, aroma and product quality studies involving Thai fish sausage [140].

Another version of RFLP, terminal restriction fragment length polymorphism (T-RFLP), in conjunction with 16S rRNA gene sequencing may be utilized with broad potential in the investigation and analysis of LAB-fermented cereals and vegetables, because it was useful in determining LAB starter cultures for gamma-Aminobutyric acid (GABA) accumulation in Myanmar fermented fish with boiled rice [141].

Other LAB identification methods, such as mass spectrometry (ESI-TOF MS) and matrix-assisted laser desorption/ionizing time-of-flight mass spectrometry (MALDI-TOF MS), can play very important roles in identifying the diversity of native lactic acid bacteria in Euro-Asian fermented cereals and vegetables, as found in selected Vietnamese and Thai fermented foods [83,142]. Metagenome shotgun sequencing and metabarcoding also have been used to detect the pathogens and virulence factors in fermented foods [143].

It should be considered that LAB starter cultures need constant enhancements, which can ensure better-defined and -characterized strains meant for cereals and vegetables fermentation, and in turn lead to the production of functional fermented food products with sensorial qualities [2].

Further, due to some local or small-scale production of the fermented products, unhygienic processing conditions are not uncommonly associated with them, thereby attracting pathogens with diverse contaminating avenues, ranging from vessels used, to raw materials and acidophilic pathogens. These health risks require cogent investigations. Other than LAB, some pathogens may be present to release harmful toxins in the fermented cereals and vegetables, such as mycotoxins, which are more common in tropical Southeast Asia.

Ashaolu [2] highlighted that natural anti-nutritional elements, such as protein inhibitors, cyanogenic glycosides, phytates, saponins, oligosaccharides, lectins and tannins, as well as biochemical residues, in most raw materials used in food fermentation should be monitored properly for their nutrient content and safety. In the same vein, several Euro-Asian traditionally fermented beverages have not been thoroughly investigated for their alcohol content levels, which the modern-day consumer is interested in.

## 7. Conclusions

There is a global demand for functional foods, and a cheap and green biotechnological means of attaining this is fermentation. Foods, especially cereals and vegetables, fermented with lactic acid bacteria offer valuable health-promoting functions to consumers. Europe and Asia have long and huge traditions in the manufacture of several LAB-fermented foods, and LAB starter culture improvement can aid in increasing the hygiene, safety and quality of fermented products.

The use of diverse microbial strains, especially LAB groups, in the production of fermented cereals and vegetables can provide socio-economic benefits and several other prospective merits. Other than culture-dependent LAB fermentation, bioinformatics coupled with DNA sequencing methods are providing clearer insights into the make-up of the LAB metatranscriptomics, in order to decipher the relationships between fermented cereals and vegetables and LAB, and their products’ sensory qualities. The small scale and local production of several Euro-Asian LAB-fermented foods imply that their characterization, safety and hygiene must be improved.

## Figures and Tables

**Table 1:** Selected lactic acid bacteria fermented cereals and vegetables in Euro-Asia.

**A1. Fermented European Cereal Products**				
**Fermented Product**	**Raw Material/Substrates**	**Country/Region**	Lactic Acid Bacteria Species Mainly Found*	**References**
Bread, Pizza, Traditional Sweet Cake (Panettone, Colomba)	Wheat sourdough	Italy, Greece, Spain, Germany, France, Belgium, Portugal, Central Europe countries	*L. acidophilus*, *L. alimentarius*, *L. amylovorus,* *L. brevis,* *L. buchneri*, *L. casei*, *L. delbrueckii*, *L. farciminis,* *L. fermentum,* *L. fructivorans*, *L. frumenti,* *L. johnsonii,* *L. mindensis*, *L. panis*, *L. paralimentarius, L. plantarum,* *L. pontis*, *L. reuteri*, *L. rossiae, L. sanfranciscensis*, *Leuc. citreum, Leuc. mesenteroides, Leuc. pseudomesenteroides, Pc. pentosaceus, W. cibaria, W. koreensis, W. confusa*	[25,26,27,28,29,30,31,32,33]
	Rye sourdough	Germany, Serbia, Bulgaria, Finland, Denmark, Norway, Sweden	*L. amylovorus, L. brevis, L. casei, L. curvatus, L. coryniformis, L. farraginis, L. frumenti, L. helveticus, L. kimchii*, *L. paralimentarius, L. paracasei, L. plantarum, L. pontis, L. panis*, *L. sanfranciscensis, L. zymae, L. uvarum, Leuc. mesenteroides, Ec. pseudoavium, Pc. acidilactici, P. pentosaceus*, *W. paramesenteroides Lc. lactis*	[37,38,39]
	Buckwheat sourdough	Ireland, Poland	*L. gramninis, L. sakei, L. fermentum, L. vaginalis, L. crispatus, L. gallinarum, W. cibaria, P. pentosaceus, Lc. holzapfelii,*	[41]
	Spelt sourdough	Belgium, Italy	*L. brevis, L. curvatus, L. fermentum, L. pentosus, L. plantarum, L. rhamnosus, Pc. acidilactici, Pc. pentosaceus, L. sanfranciscensis, Weissella spp. Leuconostoc spp.*	[40,42]
	Teff sourdough	Ireland	*L. brevis,* *L. fermentum* *L. gallinarum,* L. helveticus, *L. plantarum, L. pontis, L. sanfranciscensis,* *L. vaginalis, P. pentosaceus,* *Leuc. holzapfelii*, *W. cibaria*	[41,43]
Bread (Broa)	Maize sourdough	Portugal	*Leuconostoc spp*, *L. brevis*, *L. curvatus*, *Lc. lactis*, *E. durans*, *E. casseliflavus*, *E. faecium*, *Str. equinus*, *Str. constellantus*	[34,35,36]
Trahanas	Wheat, sheep milk	Greece, Turky, Cyprus	*L. acidophilus, L. casei, Str. lactis, Str. diacetylactis, leuc. cremoris, L. lactis*	[46,47]
Boza	Barley, oats, rye, millet, wheat, rice	Balkans (Turkey, Bulgaria)	*L. acidophilus*, *L. brevis, L. buchneri, L. coryniformis, L. fermentum,* *L. graminis, L. parabuchneri, L. casei group,* * L.* *lactis subsp. lactis, L. plantarum, L. paraplantarum,* *L.* *pentosus,* *L. raffinolactis, L. sanfranciscensis, Pc. parvulus and pentosaceus, Lc. lactis, Leuc. mesenteroides*, *Leuc. citreum, W. paramesenteroides, Oenococcus* *spp. W. confusa, W. oryzae*	[48,49,50,51,52,53]
Kaera Kiesa	Sorghum, rice, millet	Estonia		[54]
Kile	Oat flour mixed water	Estonia		[55]
Bors (Borsh)	Rye or wheat bran	Romanian	*L. amylolyticus L. fermentum, L. plantarum, L. casei, L. buchneri*.	[56]
Taar	Rye and barley, rarely also oats	Estonia		[54]
Kali	Malted cereals	Estonia		[23,54]
Kvass	Rye, barley malt	Russia	*L. casei*, *Leuc. mesenteroides*	[57,58,59]
**A2. Fermented European Vegetables Products**				
**Fermented Product**	**Raw Material/Substrates**	**Country/Region**	**Lactic Acid Bacteria Species Used**	**References**
Fermented Olives	Green olives	Spain, Portugal, Italy	*L. casei, L. coryniformis, L. pentosus, L. plantarum, Leuc. mesenteroides*, *P. pentosaceus, E. casseiflavous*	[60,61,62]
Hardaliye	Grapes/mustard seeds/cherry leaf	Turkey	*L. acetotolerans, L. brevis, L. casei, L. paracasei, L. pontis, L. sanfranciscensis, L. vaccinostercu*	[58,63,64]
Fermented Sea Fennel	Sea fennel	Italy		[65]
Turnip shreds	Rapa	Italy (Brovada)Germany (Sauerruben)	*L. plantarum, L. hilgardi; L. coryniformis, L. maltaromicus, L. viridescens, Pediococcus parvulus*	[66]
Sauerkraut	Cabbage	Europe	*L. brevis, L. plantarum, Leuc. mesenteroides,* Pediococcus *cerevisiae, E. faecalis*	[67,68]
Fermented Pickles	Cucumbers	Europe, Poland	*Lactobacillus spp., Leuconostoc spp., Pediococcus spp.*	[69]
Fermented Capers	Capers	Grece, Italy Spain	*L. plantarum, L. pentosus, L. fermentum, L. brevis, L. paraplantarum, E. faecium, P. pentosaceus*	[70]
**B1. Fermented Asian Cereal Products**				
**Fermented Product**	**Raw Material/Substrates**	**Country/Region**	**Lactic Acid Bacteria Species Used**	**References**
Amazake	Rice	Japan	*L. sakei*	[58]
Fermented Rice-Noodle (Kha nhom Jeen)	Rice	Thailand	*L. delbrueckii, L. crispatus, L. curvatus, L. lactis* ssp. *lactis, Streptococcus* spp. *Bacillus subtilis, B. licheniformis*	[71,72,73]
Sourdough	Wheat	South Korea	*L. brevis, L. paralimentarius, P. pentosaceus, W. cibaria, Leuc. citreum, W. koreensis*	[2]
*Nombonchock*	Rice	Cambodia	Various LAB *species*	[74]
*Chinese Steamed Bun*	Wheat	Thailand	*L. plantarum, L. casei*	[75]
*Comme* (Sour Rice Fermented Paste)	Rice	Vietnam	*L. casei, L. paracasei, L. acidophilus, L. plantarum*	[76]
*Bread*	Einkorn	Turkey	*L. crustorum* *, Pediococcus spp, L. brevis, L. paraplantarum, L. plantarum, L. fermentum, L. curvatus*	[77]
*Tarhana*	Wheat flour, yogurt, vegetables, spices	Turkey	*Lc. lactis, Leuc. mesenteroides, L acidophilus, E. durans, Pediococcus spp., L. delbrueckii L. paracasei*	[78]
*Tape Ketan*	Glutinous rice, Ragi	Indonesia	*Weissella spp, Pediococcus pentosaceus, Enterococcus sp*	[79]
*Selroti*	Rice-wheat flour-milk	India	*Lactobacillus spp*	[80]
**B2. Fermented Asian Vegetable Products**				
**Fermented Product**	**Raw Material/Substrates**	**Country/Region**	**Lactic Acid Bacteria Species Used**	**References**
*Naw-Mai-Dong*	Bamboo shoots	Thailand	*Leuc. mesenteroides, P. cerevisiae, Lb. plantarum, Lb. Fermentum, Lb. brevis*	[81]
*Chrourk Tror Sok, Chrourk Sapei*	Cucumber and cabbage	Cambodia	*P. pentosaceus*	[74]
Burong Mustala	Mustard	Philippines		
Pickled Vegetables (Phak-Dong)	Cabbage		*B. siamensis* B44v	[6]
Kimchi	Cabbage, green onion, pepper, ginger	Korea	*W. cibaria, W. confusa, W. koreensis*	[2]
Suan-Cai	Vegetables	China		
Pao Cai	Cabbage	China	*Leuc. mesenteroides, Lb. plantarum, Lb. casei, Lactococcus lactis*	[2]
*Pak gua Dong*	Mustard leaf	Thailand	*Lb. plantarum*	[72]
*Hom-Dong*	Red onion	Thailand		
*Jiang-Gua*	Cucumber	Taiwan		
*Dha Muoi*	Eggplant	Vietnam	*Lactobacillus fermentum, Lb. pentosus, Lb. brevis*	[82]
*Dha Muoi*	Cabbage, various vegetables	Vietnam	*Leuconostoc mesenteroides, Lb. plantatum*	[72]
*Dua Muoi*	Mustard, beet	Vietnam	*Lactobacillus fermentum, Lb. pentosus, Lb. plantarum, P. pentosaceus*	[82]
*Dua Chua* (*Dua Muoi*)	Vegetables	Vietnam	*Lactobacillus fermentum, Lb. pentosus, Lb. plantarum, P. pentosaceus, Lb. brevis*	[76]
Fermented Mustard	Mustard	Vietnam	*Lactobacillus fermentum, Lb. plantarum, Lb. brevis*	[83])
Soibum	Bamboo shoot	India		
Soidon	Bamboo shoot tips	India		
Kanji	Carrot	India		
Sunki, Tsuda-Turnip Pickle	Leaves of Otaki-turnip	Japan	*L. plantarum, L. brevis, B. coagulans, P. pentosacxeus*	[84]
Takuanzuke	Japanese radish, salt, sugar, Shochu	Japan	*L. acetotolerans L. brevis, L. casei, L. collinoides, L. curvatus, L. fructivorans, L. namurensis, L. sakei L. delbrueckii, L. fermentum, L. plantarum, L. citreum, Pc. pentosaceus, Pc. damnosus*	[84]
Sinki	Radish tap-root	India	*Lb. brevis, Lb. plantarum, Lb. fermentum*	[85]
Oiji	Cucumber, salt, water	Korea		[86]
Sayur Asin	Mustard leaves, cabbage, salt, coconut	Indonesia		
Tsukemono	Pickled vegetables	Japan	*L. brevis, L. namurensis, L. acetotolerans, L. sakei, L. curvatus, L. collinoides, L. fructivorans, L. casei, L. casei subsp. pseudoplantarum, L. delbrueckii, L. fermentum, L. plantarum, L. citreum, Pc. pentosaceus, Pc. damnosus*	[84]

* In the Table 1, we keep the names of the old classification of the genus *Lactobacillus* to avoid any confusion in the reader. However, the links for the appropriate conversions are as follows: http://lactobacillus.ualberta.ca; http://lactobacillus.uantwerpen.be; http://lactotax.embl.de/wuyts/lactotax/).
